# Superior Sagittal Sinus Thrombosis in a Young, Immunocompetent HIV Patient: A Rare Case

**DOI:** 10.7759/cureus.18625

**Published:** 2021-10-09

**Authors:** Wasey Ali Yadullahi Mir, Dhan B Shrestha, Ayusha Poudel, Vijay K Reddy, Thomas Sullivan

**Affiliations:** 1 Department of Internal Medicine, Mount Sinai Hospital, Chicago, USA; 2 Department of Medicine, Mount Sinai Hospital, Chicago, USA; 3 Intensive Care Unit, Nepal Korea Friendship Municipality Hospital, Kathmandu, NPL; 4 Department of Radiology, Mount Sinai Hospital, Chicago, USA

**Keywords:** hiv, intracranial thrombosis, venous thrombosis, headache, stroke

## Abstract

Cerebral venous thrombosis is a rare form of stroke that can present with various clinical features. In addition, it can present with nonspecific neurological features, and initial computed tomography (CT) cannot rule it out even if negative. Human immunodeficiency virus (HIV) infection is a hypercoagulable state; however, it is not much discussed. Here, we present a case of a 24-year-old immunocompetent female who presented with acute meningeal symptoms. She was managed as a case of an opportunistic central nervous system infection at initial presentation, which did not resolve her symptoms completely. Later, however, cerebral thrombosis was diagnosed, and she improved symptomatically on anticoagulants.

## Introduction

Human immunodeficiency virus (HIV) is a retrovirus that infects an individual and predominantly destroys T cells, especially CD4-positive T cells. Immune suppression is a well-known feature of the illness. However, hypercoagulability in the setting of HIV infection is not discussed widely. Venous thrombotic events are most common in HIV patients with low CD4 count and those with a long duration of illness [1]. Here, we present a case of an immunocompetent young female under treatment for HIV who presented with cerebral venous thrombosis.

## Case presentation

A 24-year-old HIV-positive female under bictegravir/emtricitabine/tenofovir alafenamide compliant to the regimen for the last two years presented to our care with complaints of a severe headache of two-day duration. She reported that the pain was throbbing in character, extremely painful (10/10 in numerical scoring system), not localized, and not relieved by taking regular analgesics. Moreover, the headache was associated with fever, neck stiffness, nausea, photophobia, and phonophobia. She reported no rashes, abnormal body movements, weakness, dizziness, or tingling sensation over the body. On neurological examination, neck rigidity and Kernig's/Brudzinski's signs were absent, and there was no focal neurological deficit. A head computed tomography (CT) revealed no acute intracranial pathology. A lumbar puncture performed on the third day of admission revealed a white blood cell (WBC) count of 70 cells/mm^3^ with 50% lymphocytes and 10% monocytes. The cerebrospinal fluid (CSF) analysis was negative for Venereal Disease Research Laboratory (VDRL), *Cryptococcus*, herpes simplex virus (HSV), and West Nile virus (WNV), and the CSF culture yielded no organisms. On suspicion of meningitis, she was empirically started on vancomycin, ceftriaxone, and acyclovir for seven days. She left against medical advice from the center and went to the emergency department (ED) of another hospital on the eighth day of treatment.

**Figure 1:**
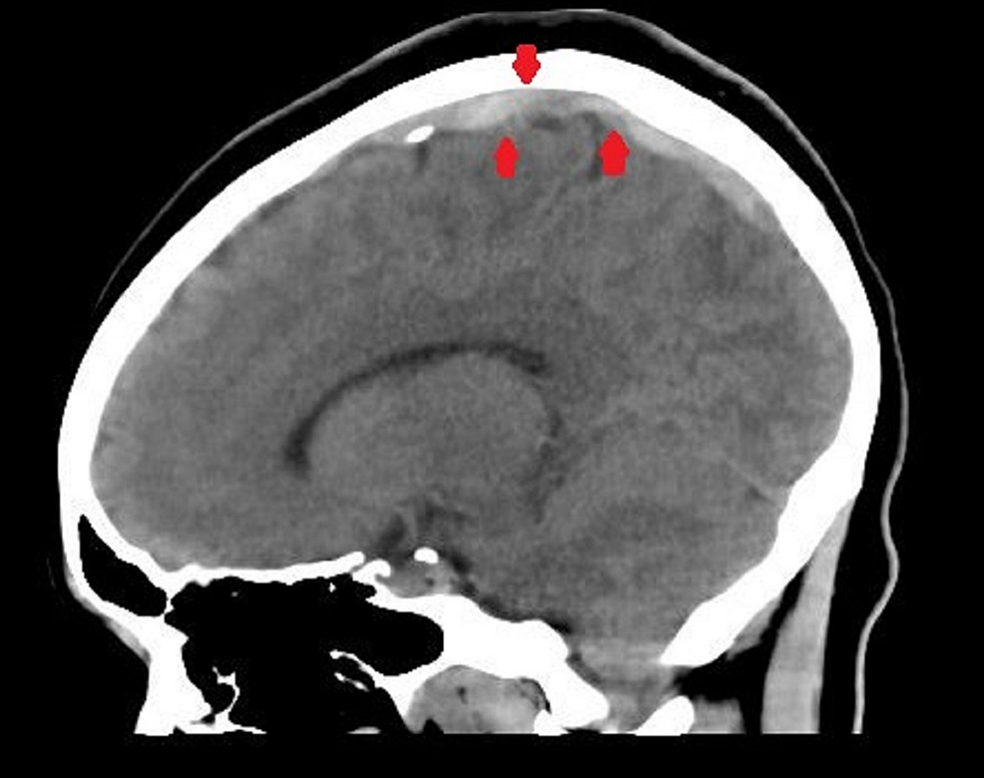
A sagittal section of brain CT scan showing a dense appearing superior sagittal sinus suspicious for thrombosis (arrows)

While presenting to the other center, her symptoms of headache, nausea, neck stiffness, and photophobia had started to subside, and she was feeling much better. A head CT was repeated, revealing the dense appearance of some cortical and larger draining veins in the superior sagittal sinus, raising suspicion for sinus thrombosis (Figure 1). A repeat lumbar puncture demonstrated a WBC count of 47 cells/mm^3^ along with 92% lymphocytes. The CSF glucose was measured to be 57 mg/dL (normal range: 40-75 mg/dL), and the total protein was 68 mg/dL (normal range: 15-40 mg/dL). The opening pressure during lumbar puncture was 150 mmH_2_O (normal range: 80-200 mmH_2_O), and the CD4 count was 1100 cells/mm^3^.

On the second day of admission at the new center, she suddenly became lethargic and had difficulty speaking with slow speech with a soft tone/whisper. Although she could move all extremities, she was slow to respond to commands. A CT venogram was performed this time and revealed a thrombus in the superior sagittal sinus and bilateral partial thrombi in the large veins draining into the sinus. Soon after the study, she developed a generalized tonic-clonic seizure that spontaneously resolved within two minutes. During the postictal period, the patient was loaded with 1000 mg levetiracetam, started on a heparin drip along with empiric acyclovir, and transferred to the step-down unit. Unfortunately, she developed a second seizure episode an hour later with a noticeable change in her mental status. She was then referred for thrombectomy of the superior sagittal sinus thrombus to a higher center. Thrombectomy, however, was deferred after discussing the risks and benefits of the procedure. In addition, she was initiated on anticoagulants. Her headache improved after five days, and she was discharged home on apixaban, levetiracetam, and topiramate.

On her follow-up three weeks later, she still had a headache, photophobia, tremors of her arms, and weakness in her right arm. A year later, her headache had resolved, and she was taken off topiramate and levetiracetam. On her last outpatient visit, one and a half years after the event, she felt fine and was only on prophylactic anticoagulation therapy.

## Discussion

Although rarely considered, HIV infection is associated with a hypercoagulable state [1]. Hypercoagulability is one of the factors, as per Virchow's triad, leading to thrombus formation. A study at the Long Island College Hospital revealed that the incidence of thrombotic events was 2.8% in HIV-positive patients [2]. Different studies have proposed several mechanisms of pathogenesis of thrombus formation in HIV infection. The various mechanisms postulated include endothelial damage due to infection [1], acquired deficiencies in protein C and S [3-6], and homocysteinemia [7]. The endothelial damages are evidenced by the detection of antiphospholipid-anticardiolipin antibodies and demonstrated microangiopathic changes [1]. The low levels of protein C have been attributed to changes in synthesis, metabolism, and the presence of low-grade disseminated intravascular coagulation (DIC) in HIV patients [5]. In addition, the increased TNF alpha during the HIV infection suppresses the active protein C, leading to impaired functioning [5]. Protein S is low due to reduction in production by endothelial cells, hepatocytes, and megakaryocytes, which are damaged due to HIV infection [5]. The deficiency in protein S may result in thromboembolic events anywhere in the body, including, but not limited to, the deep veins, vena cava, and intracranial venous or dural sinus thrombosis [6].

Thrombosis in HIV patients was found to be expected in those with opportunistic infections, with most such events in patients with CD4 counts of less than 200/mm^3^ [1]. An increased protein C deficiency is seen in HIV patients with opportunistic infections [4]. Additionally, protease inhibitors (PIs), especially indinavir, have been implicated in the development of thrombosis [1]. This fact may be explained by the evidence that TNF alpha and plasminogen activator inhibitor-1 levels are increased in patients taking PIs [8]. Our patient was taking tenofovir for the infection. A study looking into the factors associated with hypercoagulability in HIV-positive patients could not demonstrate a relationship with the CD4+ cell count but found a linear correlation with the duration of HIV infection [6].

Cerebral venous sinus thrombosis (CVST) is a rare type of stroke and is associated with an increased mortality rate [9]. The symptoms and signs of CVST are broad-ranging, and at times, diagnosing CVST entirely based on the presentation is difficult [9]. The International Study on Cerebral Vein and Dural Venous Sinus Thrombosis (ISCVT) found that superior sagittal sinus venous thrombosis was the second most common location of CVST [10]. CVST commonly presents with a headache, seizures, paresis, and papilledema in the descending order [10]. Headache is the most common presentation in patients with CVST [9]. Our patient had the chief complaint of headache during the presentation and throughout her stay at the hospital. An important caveat is that patients with CVST may present only with headaches and have no evidence of increased intracranial pressure [11]. Similar to our patient, seizures are expected during the acute phase of the disease [12].

While investigating HIV patients presenting with acute headache, a CD4 count, a complete blood count, a comprehensive metabolic panel test, a C-reactive protein test, a bacterial and fungal blood culture test, a chest X-ray, and an electrocardiogram should be done [13]. In the case of sudden headache, focal neurological deficit, altered mental status, or seizure, a cryptococcal antigen test and head imaging via CT or MRI should be done [13,14]. These investigations were performed during the presentation and were found to be nonspecific. Non-contrast CT might show a clot or stringlike density in the cerebral venous sinuses or evidence of increased intracranial pressure [9]. However, in about 30% of cases, CT might be inconclusive; thus, CVST can not be ruled out just based on CT imaging [15]. CT venography is 95% sensitive for CVST and might be helpful in the subacute or chronic presentation of CVST [16].

After the detection of CVST, the proper guideline for management is yet to be finalized. However, anticoagulation therapy with heparin has shown a decrease in poor outcomes [17]. In addition, treatment with heparin prevents clot expansion and also promotes clot resolution [18]. However, the patient was started on apixaban after a thorough discussion of risk benefits with the patient and subsequently improved while on therapy.

## Conclusions

Cerebral venous sinus thrombosis is a rare form of stroke. In hypercoagulable states such as HIV infection, sinus thrombosis should be suspected when a patient presents with neurological features or sudden headache. Early detection and management with anticoagulants show a good response for patients with cerebral venous thrombosis.
